# PRMT5-Mediated Methylation of NF-κB p65 at Arg^174^ Is Required for Endothelial CXCL11 Gene Induction in Response to TNF-α and IFN-γ Costimulation

**DOI:** 10.1371/journal.pone.0148905

**Published:** 2016-02-22

**Authors:** Daniel P. Harris, Unnikrishnan M. Chandrasekharan, Smarajit Bandyopadhyay, Belinda Willard, Paul E. DiCorleto

**Affiliations:** 1 Department of Cellular and Molecular Medicine, Cleveland Clinic Lerner Research Institute and Cleveland Clinic Lerner College of Medicine of Case Western Reserve University, Cleveland, Ohio, United States of America; 2 Department of Physiology and Biophysics, School of Medicine, Case Western Reserve University, Cleveland, Ohio, United States of America; University of Texas Rio Grande Valley, UNITED STATES

## Abstract

Inflammatory agonists differentially activate gene expression of the chemokine family of proteins in endothelial cells (EC). TNF is a weak inducer of the chemokine *CXCL11*, while TNF and IFN-γ costimulation results in potent *CXCL11* induction. The molecular mechanisms underlying TNF plus IFN-γ-mediated *CXCL11* induction are not fully understood. We have previously reported that the protein arginine methyltransferase PRMT5 catalyzes symmetrical dimethylation of the NF-κB subunit p65 in EC at multiple arginine residues. Methylation of Arg^30^ and Arg^35^ on p65 is critical for TNF induction of *CXCL10* in EC. Here we show that PRMT5-mediated methylation of p65 at Arg^174^ is required for induction of *CXCL11* when EC are costimulated with TNF and IFN-γ. Knockdown of PRMT5 by RNAi reduced *CXCL11* mRNA and protein levels in costimulated cells. Reconstitution of p65 Arg^174^Ala or Arg^174^Lys mutants into EC that were depleted of endogenous p65 blunted TNF plus IFN-γ-mediated *CXCL11* induction. Mass spectrometric analyses showed that p65 Arg^174^ arginine methylation is enhanced by TNF plus IFN-γ costimulation, and is catalyzed by PRMT5. Chromatin immunoprecipitation assays (ChIP) demonstrated that PRMT5 is necessary for p65 association with the *CXCL11* promoter in response to TNF plus IFN-γ. Further, reconstitution of p65 Arg^174^Lys mutant in EC abrogated this p65 association with the *CXCL11* promoter. Finally, ChIP and Re-ChIP assays revealed that symmetrical dimethylarginine-containing proteins complexed with the *CXCL11* promoter were diminished in p65 Arg^174^Lys-reconstituted EC stimulated with TNF and IFN-γ. In total, these results indicate that PRMT5-mediated p65 methylation at Arg^174^ is essential for TNF plus IFN-γ-mediated *CXCL11* gene induction. We therefore suggest that the use of recently developed small molecule inhibitors of PRMT5 may present a therapeutic approach to moderating chronic inflammatory pathologies.

## Introduction

Activation of endothelial cells (EC) by inflammatory substances results in stimuli-specific induction of proteins that participate in EC-leukocyte interactions and leukocyte recruitment to inflammatory foci [1]. We have previously reported that arginine methylation of transcription factors catalyzed by PRMT5 potentiates expression of the leukocyte adhesion molecules *VCAM1* and *E-selectin*, and the chemokines *CXCL10* and *CX3CL1* in response to TNF [2, 3]. PRMT5 is a member of the protein arginine methyltransferase (PRMT) family and catalyzes the covalent addition of methyl groups to the two terminal nitrogen atoms of protein-incorporated arginine. Type I and II arginine methyltransferases can add a single methyl group to arginine, producing monomethylarginine (MMA). Type I PRMT add a second methyl group to the same nitrogen atom to produce di-ω-*N*,*N*-dimethylarginine (asymmetric dimethylarginine, ADMA). In contrast, type II PRMT are capable of adding a second methyl group to the opposite terminal nitrogen atom of arginine, producing a product with methyl adducts on both terminal nitrogen residues (di-ω-*N*,*N’*-dimethylarginine; symmetrical dimethylarginine, SDMA) [4–6]. MMA, SDMA, or ADMA at a particular site may result in opposing transcriptional outcomes depending on cellular context [7, 8]. The presence of ADMA or SDMA is therefore not definitively predictive of transcriptional activity.

PRMT5 is at type II enzyme is the primary enzyme responsible for formation of SDMA in mammals [9, 10]. Addition of a methyl group to the arginine side chain increases steric bulk and hydrophobicity, and eliminates potential hydrogen bond donors, but does not alter arginine’s positive charge [9, 11, 12]. These chemical changes imparted by PRMT5 function to enhance or impede protein-substrate interactions by modulating interaction surfaces [6, 8, 13–20].

Combinations of posttranslational modifications (PTMs) such as arginine methylation, phosphorylation, and acetylation enable a “PTM code” that transduces context-specific information to the nucleus, facilitating nuanced control over transcriptional responses [21–25]. Symmetrical dimethylation of the transcription factors p53 [26–28] and NF-κB p65 [3, 29] by PRMT5 have been shown by multiple groups to enhance expression of specific genes. We reported previously that expression of *CXCL10* in response to TNF in EC requires PRMT5-catalyzed arginine methylation of the transcription factor p65 at Arg^30^ and Arg^35^ [3]. These residues are located in the proximal region of the p65 rel homology domain and are part of the p65 DNA-binding core. Arg^35^ in particular directly interacts with the 3´ subsite of the κB promoter element [30]. Methylation of these [21] residues likely enhances p65-DNA binding by facilitating hydrophobic interactions between the methyl groups and DNA base pairs [3, 29, 30].

Our previous study also identified other residues methylated by PRMT5 on p65, such as Arg^174^, a residue accessible to the cytosol that is located in a region of the rel homology domain important for mediating protein-protein interactions [31]. In the current study, we posited that p65 Arg^174^ methylation is important for mediating stimuli-specific chemokine gene expression. To test this hypothesis, we first suppressed PRMT5 levels in EC using RNAi, and stimulated the cells with TNF, IFN-γ, and TNF plus IFN-γ to identify chemokines requiring PRMT5 for induction. We discovered that PRMT5 is critical for induction of the chemokine *CXCL11* when costimulated with TNF plus IFN-γ. *CXCL11* is transcribed upon stimulation by EC, astrocytes, monocytes, neutrophils, and keratinocytes [32]. Ligation of CXCL11 to the classical CXCR3 receptor enriched on activated Th1-type (type-1 helper) CD4^+^ and CD8^+^ cytotoxic T-lymphocytes (CTL), CD4^+^ and CD8^+^ memory cells, natural killer (NK), natural killer T cells (NKT), dendritic cells, and some B cells results in recruitment of leukocyte populations to inflamed sites [33–35]. CXCL11 further increases the polarity of lymphocytes at inflammatory lesions by antagonizing the CCR3 receptor enriched on Th2-type lymphocytes [36]. CXCL11 participates in numerous pathologies including atherosclerosis, organ transplant, inflammatory arthritis, inflammatory bowel disease, psoriasis, asthma, hematopoietic malignancies, and responses to infection [33, 34, 37, 38].

Additional characterization of the role of PRMT5 in the induction of *CXCL11* revealed a requirement for methylation of p65 at Arg^174^. Together with our previous report [3], our results show that arginine methylation of p65 residues by PRMT5 comprises a critical aspect of the PTM code governing the specificity of inflammatory chemokine gene expression.

## Materials and Methods

### Ethics Statement

HUVEC are isolated from umbilical collected by MetroHealth Hospital and Hillcrest Hospital. The cords are not linked to any patient identification, and isolated EC are pooled. The Cleveland Clinic Foundation Institutional Review Board has confirmed that our use of discarded, de-identified human tissue is exempt from review under National Institutes of Health guidelines.

### Cell Culture and Reagents

Primary human EC were isolated from human umbilical cords [39]. Cells from multiple individuals were pooled and used at early passages (P2-4). Cells were cultured in MCDB 107 medium (Sigma Life Sciences) supplemented with 15% FBS (Atlas Biologicals), 150 μg/ml endothelial cell growth supplement (ECGS), and 90 μg/ml heparin (Sigma-Aldrich). Recombinant TNF-α (R&D Systems) was used at 2 ng/ml. Recombinant IFN-γ (R&D Systems) was used at 450 U/ml. Cells were serum starved in MCDB media for 3 hours prior to the addition of agonists. Antibodies used in immunoblotting were anti-PRMT5 (Millipore 07–405), anti-p65 (Millipore 06–418), anti-dimethyl-arginine symmetric (SYM10, Millipore, 07–412), anti-CXCL11 (R&D Systems MAB672), anti-MCP1 (Cell Signaling Technology #2027), and anti-GAPDH (Cell Signaling Technology #2118). ChIP antibodies included anti-FLAG M2 (Sigma F1804) and anti-SDMA (SYM10). Additional details of the antibodies used are provided in S1 Table.

### Transfection, Constructs, and RNAi

Cells were transfected 12–18 h following passage at 70–80% confluency. At this time complete cell media containing 15% FBS was removed, and replaced with a transfection mixture containing Targefect F-2 (5 μl/ml) and peptide enhancer (5 μl/ml) (Targeting Systems) in serum-free Dulbecco’s modified eagle’s medium (DMEM; Sigma-Aldrich). siRNA was transfected at 50 nM, whilst cDNA was transfected at 250 ng/ml. The transfection mixture was incubated with the cells at 37°C for 4 h, at which point it was removed and replaced with complete growth media containing serum. Cells were maintained in this media for 24–48 h to allow for protein expression or knockdown. Details of our wild type p65, p65 Arg^174^Ala and Arg^174^Lys cDNA constructs can be found in our previous publication [3]. In brief, human p65 cDNA (Addgene construct 21966) was inserted into pCDNA3 with an N-terminal FLAG epitope tag (AA: DYKDDDDK) in frame with the start codon and expressed under the control of the CMV promoter. Arg^174^ was mutated using the PCR-based GeneArt Mutagenesis System (Life Technologies). We repeated all experiments using two different siRNA sequences for each target to minimize the potential of off-target effects. siRNAs complementary to the PRMT5 coding sequence were sense 5´-GAGGGAGUUCAUUCAGGAAUU-3´, and PRMT5 3´ UTR, sense 5´-GCUCAAGCCACCAAUCUAUUU-3´. siRNAs targeting the NF-κB p65 3´ UTR were sense 5´-GGAUUCAUUACAGCUUAAUUU-3´ and sense 5´-GCUCUUUCUACUCUGAACUUU-3´. All siRNAs were designed using the Whitehead Institute siRNA design tool (http://sirna.wi.mit.edu/) and synthesized by Ambion containing the Silencer Select modifications [3]. Nontargeting Silencer Select siRNA was used as a control (Ambion).

### Immunoblotting

Lysates for immunoblots were prepared by addition of 2X Laemmli buffer. After boiling for 5 min, proteins were resolved on 10% bis-tris acrylamide gels by SDS-PAGE, and transferred to PVDF membranes using a semi-dry transfer apparatus (Bio-Rad). Membranes were blocked with either 5% nonfat milk in 1X TBST or protein-free TBS blocking buffer (Pierce). Primary antibody incubation occurred overnight at 4°C with gentle agitation. Multiple washes were performed with 1X TBST. Secondary antibodies were HRP-conjugated. Chemiluminescence was activated using Western Lightning Plus ECL Substrate (PerkinElmer). All immunoblots were quantified via the Image Studio Lite program (LI-COR Biosciences) and normalized to a loading control.

### RNA Purification and Quantitative Real Time PCR

Cells were lysed in 350 μl of Buffer RLT containing 1% 2-mercaptoethanol (Sigma-Aldrich) per each well of a 6-well plate following agonist exposure. Total RNA was isolated with the RNeasy kit (Qiagen). SuperScript First-Strand reagents (Life Technologies) were used to produce cDNA from 1 μg of RNA. The resulting cDNA was diluted 3-fold with water. qRT-PCR was performed using Fast SYBR Green Reagent (Life Technologies) using an ABI StepOnePlus machine (Life Technologies) with 40 cycles of amplification. Primers were separated by an intron on the corresponding gDNA and amplified 100–150 base pairs. Results were normalized to GAPDH according to the –ΔΔCt method. Primer sequences are provided in S2 Table. Amplification products were validated with sequencing, and analysis of amplification and melt curves.

### Quantitative Mass Spectrometry

p65 and PRMT5 siRNAs were transfected into EC. After 40 hours, cells were serum starved and treated with TNF and IFN-γ then lysed in 1X RIPA. FLAG-p65 was immunoprecipitated using anti-FLAG antibodies. Proteins were separated by PAGE and the gel was silver-stained. p65 bands were excised and digested in-gel with trypsin overnight at room temperature. Fragments were assessed using capillary column LC-tandem mass spectrometry using a self-packed 9 cm x 75 μm inner diameter Phenomenex Jupiter C18 reversed-phase capillary chromatography column and Finnigan LTQ (linear trap quadrapole) ion trap mass spectrometer (ThermoFinnigan). Collision-induced dissociation (CID) spectra were analyzed using the NCBI human non-redundant protein database. Targeted experiments were also performed involving selected reaction monitoring (SRM) of specific p65 peptides, including unmodified and arginine methylated forms. Chromatograms derived from these data were assessed in relation to known fragmentation patterns. Peak areas were used to quantitate the extent of methylation.

### Chromatin Immunoprecipitation and Sequential ChIP (Re-ChIP)

Cells for ChIP were cultured in 10 cm dishes. The ChIP protocol was followed according to the instructions included in the ChIP kit (Millipore 17–295). Crosslinked protein-DNA complexes were formed by incubating the cells with 1% formaldehyde (Sigma-Aldrich) in MCDB media for 10 min at 37°C. Following sonication, immunoprecipitation, and crosslink reversal the immunoprecipitated DNA was purified with the PrepEase DNA Cleanup kit (Affymetrix). In the Re-ChIP experiments the complexes from the initial IP (anti-FLAG) were eluted at 37°C for 30 minutes with 10 mM DTT. Following centrifugation the supernatant was removed to a fresh tube and diluted 20 times in Re-ChIP buffer (1% Triton X-100, 2 mM EDTA, 150 mM NaCl, 20 mM Tris-HCl (pH 8.1). The second IP antibody (anti-SDMA) or antiserum was then added, followed by crosslink reversal and DNA purification as outlined above. DNA products from the immunoprecipitation were quantified by qRT-PCR relative to input. Primers were designed to amplify chemokine proximal promoters containing κB site(s) and provided in S3 Table.

### Statistical Analysis

All experiments were repeated with an *n* = 3–4 and are presented as mean ± standard error of the mean (SEM). One- or two-way analysis of variance (ANOVA) were performed. Pairwise comparisons were assessed with Student’s t-test, and corrected with Bonferroni post hoc tests. A *p* value of ≤ 0.05 was considered significant.

## Results and Discussion

In our previous publication, we identified that symmetrical dimethylarginine (SDMA) formation catalyzed by PRMT5 on Arg^30^ and Arg^35^ of p65 is necessary for induction of *CXCL10* by TNF in EC [3]. Methylation of these residues is part of a signaling mechanism enabling transcription of the chemokines *CXCL10* and *CX3CL1*, and not other pro-inflammatory chemokines, such as *CXCL8/IL-8* or *CCL2/MCP1*. We also discovered methylation at other p65 arginine residues, such as Arg^174^, and postulated that arginine methylation of this residue may be necessary to induce other pro-inflammatory chemokines in a stimulus-dependent manner. In particular, we explored the effects of EC costimulation by TNF plus IFN-γ as this combination of agonists synergistically induces *CXCL10* [40–42] and *CXCL11* [43–45] expression. Coincident stimulation of EC with TNF and IFN-γ is physiologically relevant as both factors are simultaneously present in localized regions of inflammation under pathological conditions, such as atherosclerosis [46].

**Fig 1:**
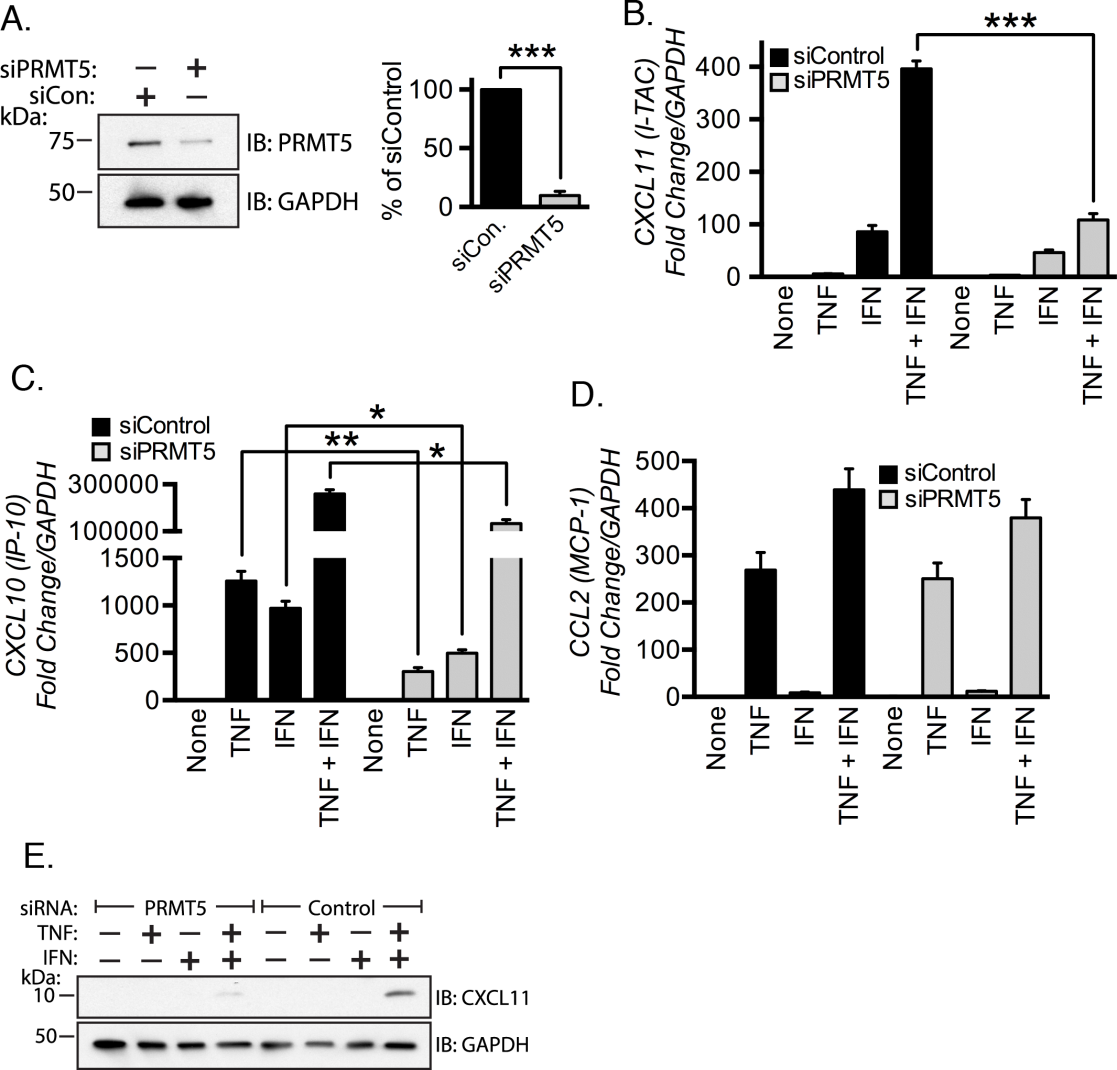
PRMT5 promotes expression of *CXCL11* in EC costimulated with TNF and IFN-γ. **(A)** PRMT5-specific siRNA depleted protein levels in endothelial cells (EC) by ~90% as measured by immunoblotting (left panel) and densitometry (right panel). Expression of the chemokines *CXCL11* **(B)**, *CXCL10* **(C)** and *CCL2* **(D)** were measured by real-time PCR of RNA isolated from EC transfected with the indicated siRNAs and subsequently stimulated with TNF, IFN-γ, or TNF plus IFN-γ for 3 hours. An immunoblot showing CXCL11 protein expression in PRMT5 or control siRNA-transfected cells after 3 h of stimulation is shown in **(E)**. *, *p* < 0.05; ***, *p* < 0.005; error bars represent S.E. (*n* = 3–4).

We tested our hypothesis by measuring chemokine mRNA expression levels in control versus PRMT5-depleted EC. Cells were serum-starved for 48 h post siRNA transfection and then stimulated with TNF, IFN-γ, and TNF plus IFN-γ. Total RNA was isolated and real-time PCR was performed to measure chemokine mRNA levels. Knockdown of PRMT5 efficiently suppressed PRMT5 protein levels by greater than 90% (Fig 1A, *p* < 0.001). We found that transcription of *CXCL11* mRNA was significantly blunted in PRMT5-depleted EC in response to TNF plus IFN-γ costimulation (~30% of the mRNA elicited from the control cells, *p* < 0.05, Fig 1B). PRMT5-depletion did not significantly alter *CXCL11* mRNA abundance in response to IFN-γ stimulation alone (*p* > 0.05). We observed a significant decrease in *CXCL10* mRNA induction when both agonists were presented individually, and in combination (Fig 1C, all *p* < 0.05). TNF plus IFN-γ-mediated induction of *CCL2* (Fig 1D) was not significantly affected by PRMT5 depletion. We verified a positive role for PRMT5 in *CXCL11* induction at the protein level by immunoblot (Fig 1E). These results indicate that PRMT5 is necessary for *CXCL11* induction in response to TNF and IFN-γ costimulation.

**Fig 2:**
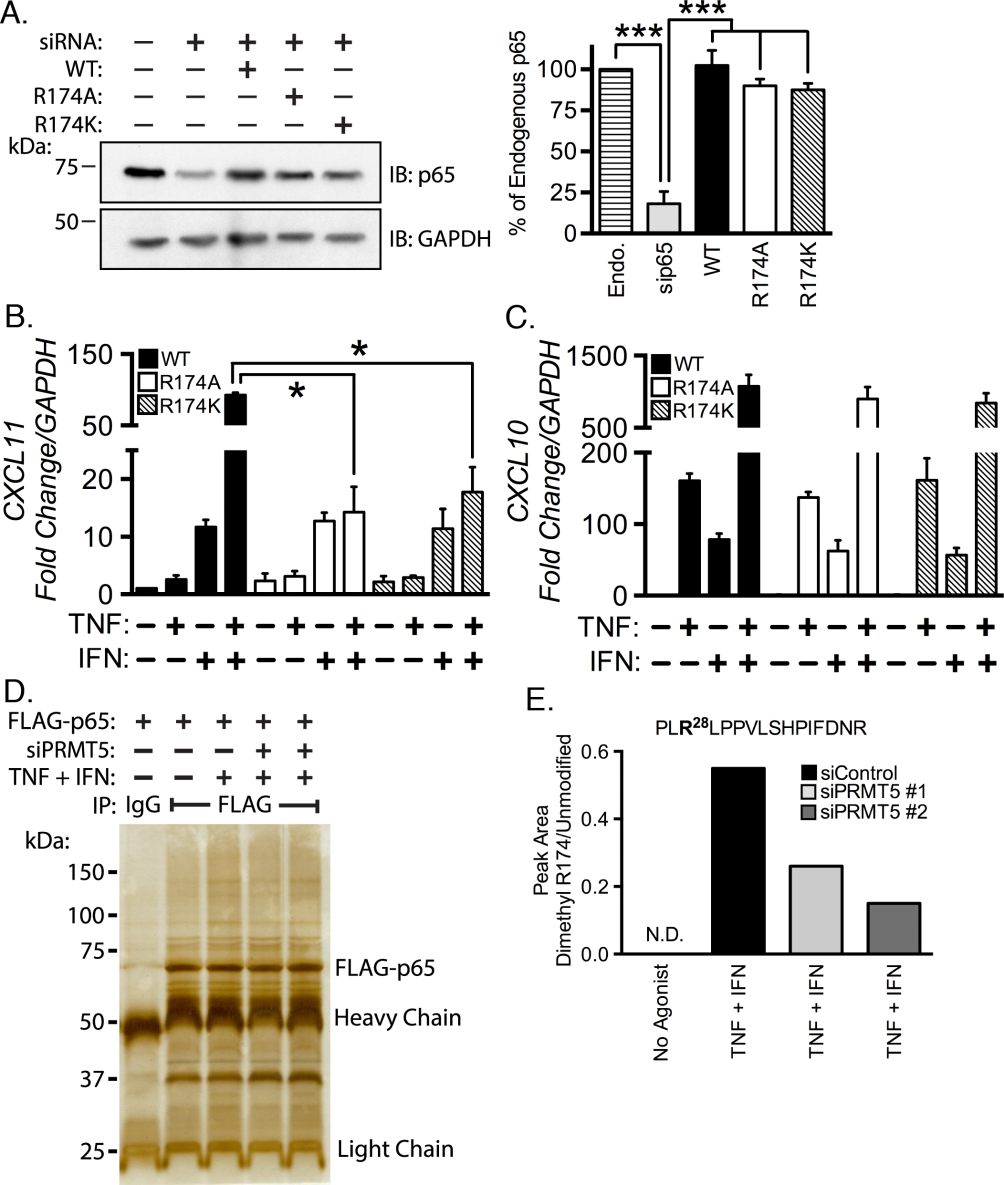
TNF and IFN-γ mediated *CXCL11* induction requires PRMT5 catalyzed p65 dimethylation at Arg^174^. siRNA targeting the 3´ UTR of p65 was cotransfected in EC with cDNA encoding wild type, Arg^174^Ala, or Arg^174^Lys p65. Knockdown of endogenous p65 and reconstitution with p65 constructs is shown by immunoblot and densitometric analysis **(A)**. EC reconstituted with wild type and p65 mutants were treated with the indicated pro-inflammatory agonists for 3 hours followed by RNA collection and real-time PCR analysis to determine the mRNA levels of *CXCL11* **(B)** and *CXCL10* **(C)**. In **(D)**, FLAG-p65 was immunoprecipitated from EC transfected with two different PRMT5 siRNAs (sequence 1, lane 4; sequence 2, lane 5) following stimulation with TNF and IFN-γ for 30 minutes. Immunoprecipitates were separated by PAGE and the gel was silver-stained. FLAG-p65 bands were excised for mass spectrometric analysis. **(E)** Mass spectrometric quantitation of the peak area of dimethylated Arg^174^ present in the tryptic ^172^PL**R**LPPVLSHPIFDNR^185^ peptide relative to the unmodified peptide. The 28 in the peptide sequence refers to the +28 Da mass shift consistent with dimethylation (2 x 14 Da) of Arg^174^.*, *p* < 0.05; ***, *p* < 0.005; error bars represent S.E (*n* = 3).

We then asked whether Arg^174^ must be present in order to transactivate *CXCL11*. We cotransfected siRNA targeting the 3´ UTR of p65 along with cDNA encoding wild type p65, p65 Arg^174^Ala, or p65 Arg^174^Lys. PRMT5 is incapable of methylating alanine and lysine residues [5, 47–49]. Transfection of p65 siRNA depleted ~80% of p65, and reconstitution of wild type or either Arg^174^ point mutant recapitulated endogenous levels of p65 (Fig 2A, quantified in right panel). EC reconstituted with p65 mutants were then stimulated with TNF, IFN-γ, or TNF plus IFN-γ, and *CXCL11* mRNA levels were measured with quantitative real-time PCR. Wild type p65 reconstituted-EC costimulated with TNF and IFN-γ showed synergistic *CXCL11* induction. The synergistic induction of *CXCL11* by TNF or IFN-γ in wild type-reconstituted EC was ~92 fold increased over unstimulated cells. Importantly, *CXCL11* induction was dramatically reduced in cells reconstituted with either Arg^174^Ala (~14 fold increase over unstimulated, *p* < 0.05) or p65 Arg^174^Lys (~18 fold increase over unstimulated, *p* < 0.05; Fig 2B). *CXCL11* mRNA levels were not significantly different in wild type or Arg^174^ mutant reconstituted EC upon stimulation with TNF or IFN-γ in isolation. These results suggest that p65 Arg^174^ is critical for the synergistic induction of *CXCL11* in response to TNF and IFN-γ costimulation. In contrast, expression of *CXCL10* in cells expressing p65 WT or either Arg^174^ mutant stimulated with either agonist alone or in combination was comparable to EC expressing wild type p65 (Fig 2C). This finding was unsurprising given our previous findings that methylation of Arg^30^ and Arg^35^, but not Arg^174^, is necessary to transactivate *CXCL10* [3].

We next sought to determine whether Arg^174^ methylation levels are modulated in response to costimulation with TNF and IFN-γ. We introduced wild type FLAG-p65 cDNA along with control or PRMT5 siRNA. After 40 hours, we stimulated the cells, prepared lysates, and immunoprecipitated p65 using the FLAG tag. IP products were separated using PAGE and silver-stained (Fig 2D). The 65 kDa bands were excised, digested with trypsin, and fragmented by collision induced dissociation. A mass spectra of the ^172^PL**R**LPPVLSHPIFDNR^185^ peptide containing dimethylarginine at Arg^174^ is provided in S1 Fig. Peptides were also subjected to mass spectrometric selected reaction monitoring experiments to quantitate relative levels of arginine methylation. We found that p65 Arg^174^ dimethylation was undetected under unstimulated conditions, but was present in ~0.6 percent of the ^172^PL**R**LPPVLSHPIFDNR^185^ peptide (Fig 2E). We did not detect monomethylation with or without treatment with TNF and IFN-γ. Additionally, we determined that Arg^174^ dimethylation was catalyzed by PRMT5, as depletion of PRMT5 using two different sequences in TNF plus IFN-γ treated cells diminished Arg^174^ methylation to ~50% and ~33% of the control transfected, costimulated levels (Fig 2E), respectively. Together, these findings suggest a requirement for PRMT-mediated p65 Arg^174^ methylation in the expression of *CXCL11*, but not *CXCL10*, when EC are costimulated with TNF and IFN-γ. We do not contend that Arg^174^ methylation is the only p65 PTM necessary for *CXCL11* activation, as other modifications such as phosphorylation, may also contribute to *CXCL11* induction.

**Fig 3:**
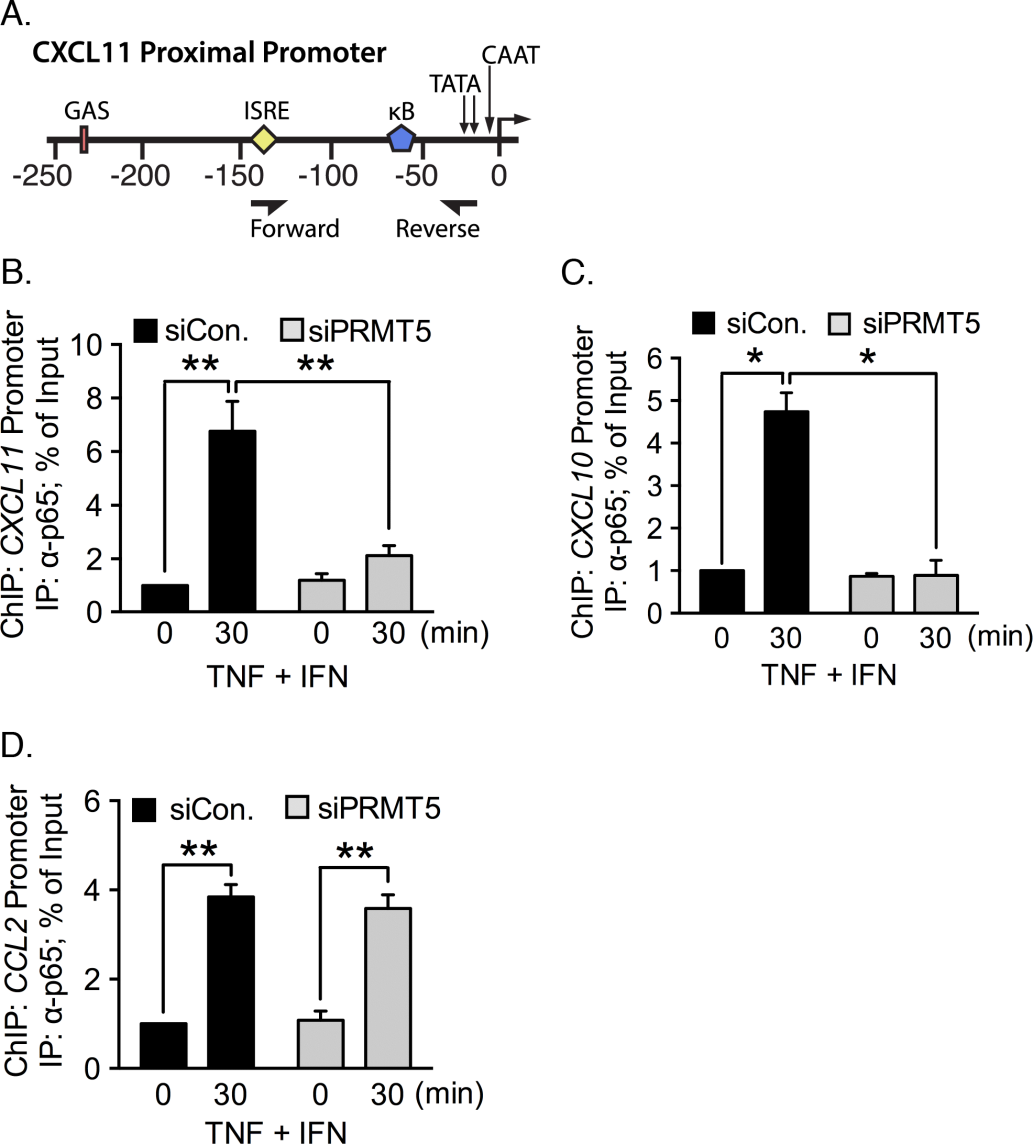
PRMT5 knockdown reduces association of p65 with the *CXCL11* promoter. The location of major regulatory elements and primer binding sites used for the chromatin immunoprecipitation (ChIP) assay of the *CXCL11* promoter are illustrated in **(A)**. IFN-γ response elements include the gamma interferon activation site (GAS) and interferon-sensitive response element (ISRE). The major TNF responsive element is the NF-κB binding site. ChIP assays were performed to assess p65 binding with the proximal promoter encompassing the κB binding site(s) of *CXCL11* **(B)**, *CXCL10* **(C)**, and *CCL2* **(D)** in PRMT5-intact or -depleted EC stimulated with TNF plus IFN-γ for 30 minutes. *, *p* < 0.05; **, *p* < 0.01; error bars represent S.E. (*n* = 3).

We next performed ChIP to determine whether PRMT5 is obligatory for p65 association with the *CXCL11* promoter following TNF plus IFN-γ costimulation. We immunoprecipitated chromatin from PRMT5-intact or -depleted EC using an anti-p65 antibody. We then amplified *CXCL11* promoter fragments using primers encompassing the κB site (Fig 3A). p65 was found to be associated with the *CXCL11* promoter upon TNF plus IFN-γ exposure (~7 fold increase) but PRMT5-depletion decreased p65 binding to the *CXCL11* promoter significantly (~2 fold increase, *p* < 0.01) as compared with the unstimulated condition (Fig 3B). We were unable to detect p65 on the *CXCL10* promoter in PRMT5-depleted EC (Fig 3C). In contrast, p65 association with the *CCL2* (*MCP1*) promoter following costimulation was unaffected by PRMT5 knockdown (Fig 3D), in line with our observations that PRMT5 is not involved in *CCL2* expression in response to TNF, IFN-γ, or TNF plus IFN-γ stimulation (Fig 1D). These results reveal that PRMT5 activity is critical for p65 recruitment to the *CXCL10* and *CXCL11* promoters when EC are costimulated with TNF and IFN-γ.

**Fig 4:**
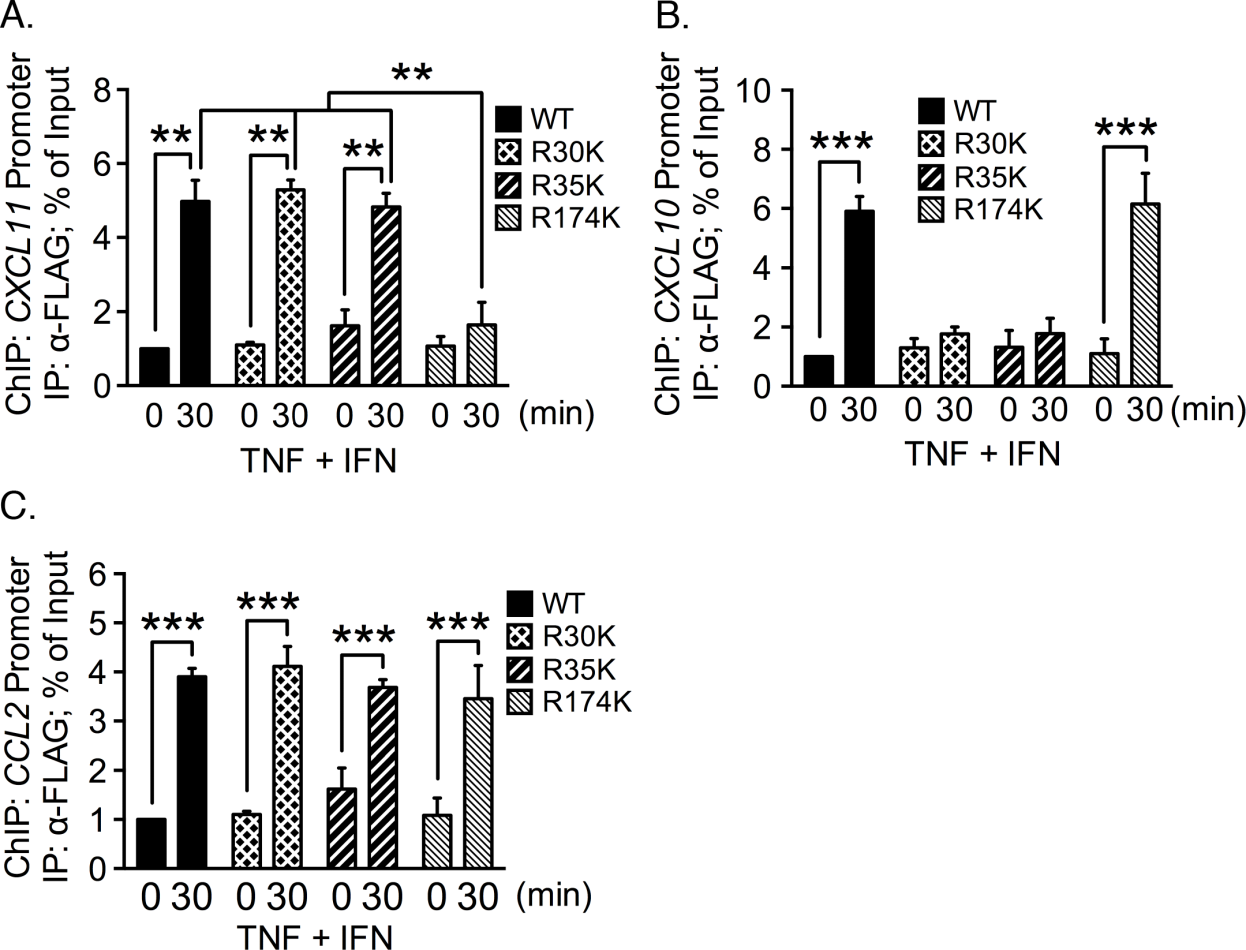
Mutation of Arg^174^ to lysine abrogates p65 association with the *CXCL11* promoter. Endogenous p65 was depleted with siRNA targeting the 3´ UTR of p65 in EC. p65 was reconstituted in these cells by transfecting cDNA encoding FLAG-tagged wild type p65 or the FLAG-tagged Arg^174^Lys mutant. Following cell stimulation, the protein-DNA complexes were immunoprecipitated with an anti-FLAG antibody. Enrichment of the *CXCL11* **(A)**, *CXCL10* **(B)**, and *CCL2* **(C)** promoters by the IP was quantified by real-time PCR. *, *p* < 0.05; ***, *p* < 0.005; error bars represent S.E. (*n* = 3).

Additional ChIP assays were performed to ascertain whether p65 Arg^174^Lys is recruited to the *CXCL11* promoter. Mutation of arginine-to-lysine is a conservative mutation that preserves the positive charge at amino acid 174 but precludes the possibility of methylation by PRMT5. Cells were cotransfected with siRNA targeting the 3´ UTR of p65 along with cDNA encoding either FLAG-tagged wild type p65 or Arg^174^Lys. DNA-protein complexes were immunoprecipitated with anti-FLAG antibody and probed with primers that amplify the *CXCL11* promoter. We detected a significant increase in the association of wild type p65 with the *CXCL11* promoter following costimulation (Fig 4A). However, the level of *CXCL11* promoter immunoprecipitated from cells reconstituted with p65 Arg^174^Lys was not statistically different from unstimulated cells. In contrast, *CXCL10* (Fig 4B) and *CCL2* (Fig 4C) promoter fragments were enriched equally well in costimulated cells expressing either wild type p65 or Arg^174^Lys p65. We conclude that there is a requirement for p65 Arg^174^ for the expression of *CXCL11*, but not *CXCL10* or *CCL2*.

**Fig 5:**
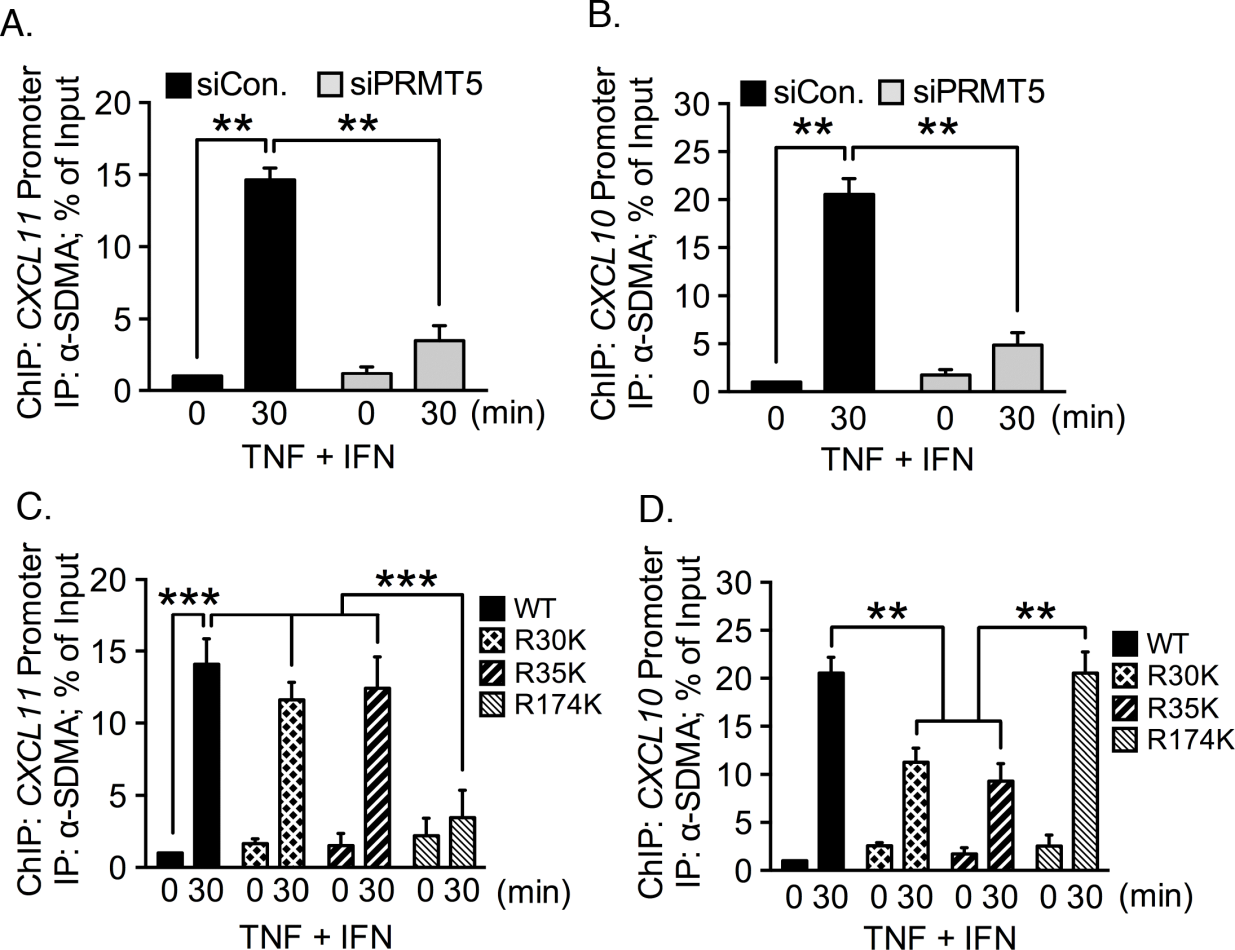
PRMT5 depletion diminishes detection of SDMA-containing proteins associated with the *CXCL11* promoter. EC were transfected with PRMT5-specific or control siRNA and stimulated with TNF plus IFN-γ. ChIP was performed using an antibody specific for SDMA. Enrichment of the *CXCL11* **(A)** and *CXCL10* **(B)** promoters was quantified by qRT-PCR. **(C-D)** ChIP was performed on EC reconstituted with FLAG-tagged wild type p65 or the indicated p65 mutants from TNF plus IFN-γ stimulated EC. Protein-DNA complexes were immunoprecipitated with anti-FLAG antibody. Enrichment of the *CXCL11* **(C),** and *CXCL10* **(D)** promoters was quantified by qRT-PCR. **, *p* < 0.01; error bars represent S.E. (*n* = 3).

In our previous publication we showed that p65 contains SDMA catalyzed by PRMT5 [3]. With that information in mind, our next question in the current project asked whether we could detect SDMA-containing proteins associated with the *CXCL11* promoter in TNF plus IFN-γ costimulated cells. We immunoprecipitated chromatin from PRMT5 siRNA-transfected, costimulated cells using an anti-SDMA antibody. We were able to detect a robust enrichment of the *CXCL11* promoter following stimulation, but this enrichment was lost in the absence of PRMT5 (Fig 5A). Results were similar when we assayed the immunoprecipitated DNA for the *CXCL10* promoter: we were able to detect SDMA-associated *CXCL10* promoter after stimulation in the endogenous condition, but not when PRMT5 was depleted (Fig 5B). We therefore concluded that SDMA-catalyzed by PRMT5 is associated with both the *CXCL10* and *CXCL11* promoters after simultaneous exposure to TNF and IFN-γ.

We next sought to determine whether arginine methylation of p65 amino acid 174 is necessary for *CXCL11* induction. We reconstituted EC with wild type, Arg^30^Lys, Arg^35^Lys, and Arg^174^Lys p65, and performed ChIP with an anti-SDMA antibody. Results indicated that the *CXCL11* promoter was pulled down when wild type p65 and the p65 Arg^30^Lys and Arg^35^Lys mutants were expressed in costimulated EC (Fig 5C). When p65 Arg^174^Lys was reconstituted, the *CXCL11* promoter levels were not statistically different from the unstimulated condition. This suggests that p65 with lysine at position 174 does not associate with the *CXCL11* promoter.

The ChIP immunoprecipitates were also probed for the presence of the *CXCL10* promoter. We found that the *CXCL10* promoter was enriched from both wild type and Arg^174^Lys reconstituted cells upon costimulation (Fig 5D, *p* < 0.005). When the Arg^30^Lys and Arg^35^Lys mutants were expressed, levels of *CXCL10* promoter dropped to ~50% of the levels observed with the wild type and Arg^175^Lys mutant (Fig 5D, *p* < 0.005). These results are consistent with our previous finding that methylation of p65 Arg^30^ and Arg^35^ are necessary for *CXCL10* induction in EC. Additionally, these results indicate that Arg^30^ and Arg^35^ may be methylated independently, as mutation of either Arg^30^ or Arg^35^ to lysine does not reduce methylation of the other residue.

**Fig 6:**
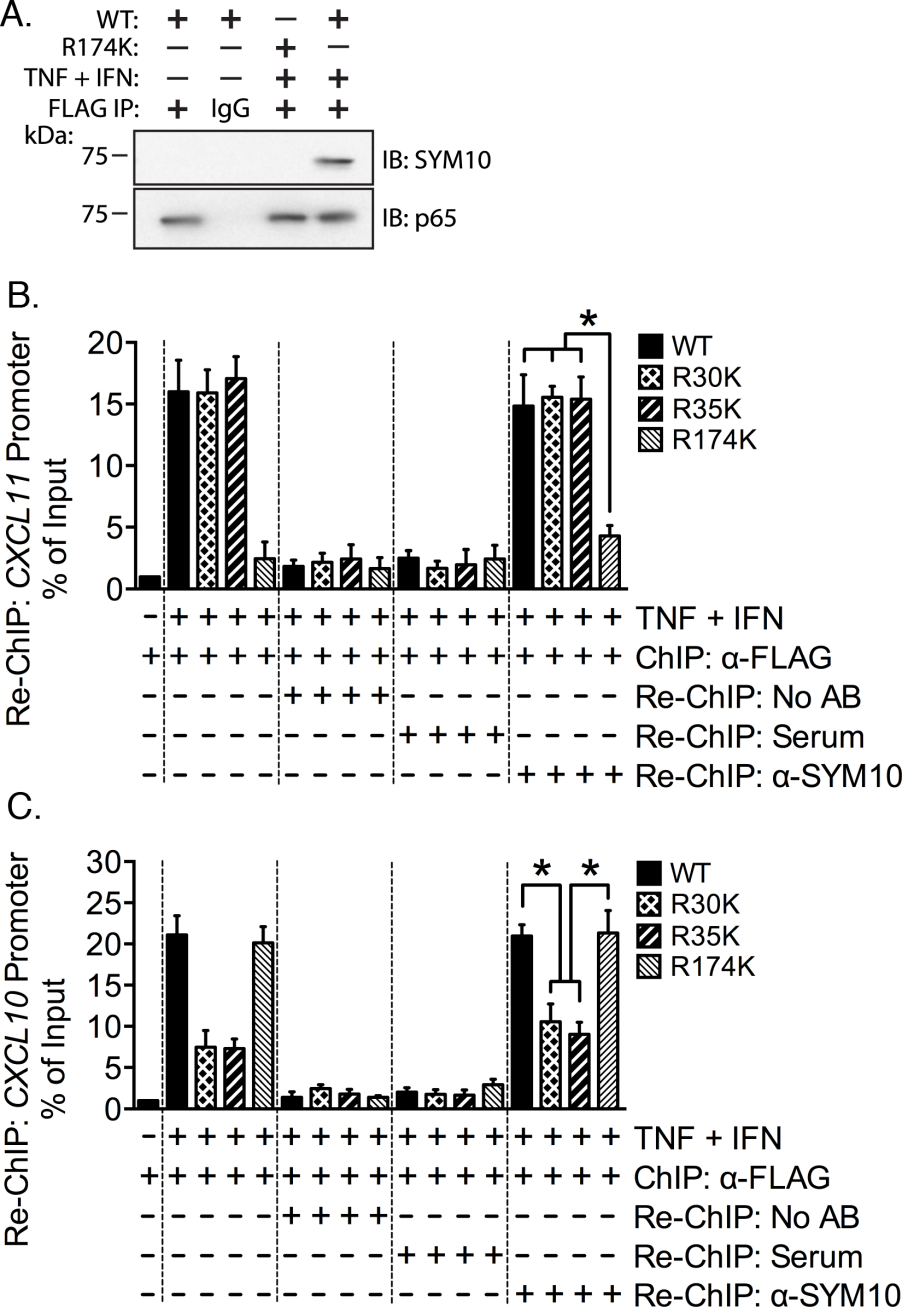
p65 Arg^174^ methylation is present on the *CXCL11* promoter following costimulation. **(A)** EC were depleted of endogenous p65 and reconstituted with wild type or Arg^174^Lys mutant p65. Cells were stimulated with TNF plus IFN-γ for 30 minutes and lysed in RIPA buffer. Wild type and mutant p65 was immunoprecipitated using the FLAG epitope or IgG. Immunoprecipitates were immunoblotted for p65 and for the SDMA modification. **(B-C)** Cells were reconstituted with *N*-FLAG wild type, Arg^30^Lys, Arg^35^Lys, and Arg^174^Lys p65 constructs, and simultaneously exposed to TNF and IFN-γ. ChIP was performed using anti-FLAG antibody. Sequential ChIP (Re-ChIP) was carried out with addition of normal rabbit serum or anti-SYM10 antibody. Following crosslink reversal, DNA was purified and promoter fragment content was quantitated using qRT-PCR relative to input.

To confirm that the SDMA antibody is detecting methylation at p65 Arg^174^ we reconstituted wild type and Arg^174^Lys mutants in EC, and used the FLAG epitope to immunoprecipitate p65 proteins, which where then probed with anti-SDMA antibody (SYM10; Fig 6A). We are able to detect arginine methylation in response to TNF plus IFN-γ only when wild type p65 is present (lane 4), but not when the Arg^174^Lys was transfected (lane 3). These results indicate that the symmetrical dimethylation of p65 detected by the anti-SDMA antibody is likely modification of Arg^174^.

We next sought to assess whether p65 and SDMA are detected on the same fragments of the *CXCL11* promoter. We utilized a sequential ChIP (Re-ChIP) approach where we reconstituted wild type and point mutant p65, then immunoprecipitated p65 using the FLAG-tag. After removal of the anti-FLAG antibody, we immunoprecipitated the protein-chromatin complexes a second time using the anti-SDMA antibody. The *CXCL11* promoter was present at background levels in the absence of cell costimulation with TNF and IFN-γ following immunoprecipitation of wild type p65 (Fig 6B, bar 1). We found *CXCL11* promoter enrichment when wild type, Arg^30^Lys, and Arg^35^Lys were expressed in costimulated cells where p65 was immunoprecipitated with the anti-FLAG antibody (Fig 6B, bars 2–4). In contrast, we detected the *CXCL11* promoter when Arg^174^Lys p65 was expressed only at background levels (bar 5).

We included a no antibody control to assess whether the anti-FLAG antibody was successfully dissociated from the chromatin during the Re-ChIP procedure. We did not detect the *CXCL11* promoter with this control (Fig 6B, bars 6–9), indicated that we were able to remove the antibody with buffer exchange. Following buffer exchange, we proceeded with the Re-ChIP portion of the experiment, using either the anti-SDMA antibody (SYM10) or normal rabbit serum, as SYM10 is presented in antiserum format (bars 10–13). We did not enrich the *CXCL11* promoter in the Re-ChIP stage of the experiment using the serum regardless of whether wild type or any of the mutants were present.

Enrichment of the *CXCL11* promoter was detected equal to roughly 15% of the input material when wild type, Arg^30^Lys, and Arg^35^Lys p65 were present (bars 14–16). Crucially, we found that the level of *CXCL11* was significantly diminished when Arg^174^Lys was expressed to ~5% of input (*p* < 0.05; bar 17). In full, these results indicate that the methylation associated with the *CXCL11* promoter is predominately symmetrical dimethylation of p65 at Arg^174^. Arginine methylation of other p65 arginine residues, such as Arg^30^ or Arg^35^, is only a minor component of p65 methylation associated with this promoter under TNF plus IFN-γ costimulated conditions.

We also quantitated the presence of the *CXCL10* promoter in this experiment (Fig 6C). p65 is not associated with the promoter in the absence of TNF and IFN-γ (bar 1). A robust increase in promoter association was observed when wild type and Arg^174^Lys were expressed in stimulated cells and immunoprecipitated with the anti-FLAG antibody (~20% of input). When Arg^30^Lys and Arg^35^Lys were present, *CXCL10* promoter was enriched to ~7% of input (*p* < 0.05; bars 2–5). Only background levels of *CXCL10* promoter were found following buffer exchange to remove the anti-FLAG antibody (bars 6–9). Levels of *CXCL10* promoter were also at background when rabbit serum was applied in the Re-ChIP. Quantitation of the promoter showed following Re-ChIP with the SYM10 antibody showed that wild type and Arg^174^Lys p65 pulled down *CXCL10* promoter equal to ~20% of input, but that Arg^30^Lys and Arg^35^Lys mutant expression was associated with lower quantities of the promoter, ~8–10% of input (*p* < 0.05; bars 14–17). These results are consistent with our previous findings that methylation of Arg^30^ and Arg^35^ is necessary for *CXCL10* induction, but that Arg^174^ methylation is not required [3]. It is interesting to note that mutation of either Arg^30^ or Arg^35^ to lysine reduces, but does not eliminate, detection of methylated p65 associated with this promoter, supporting a model where methylation of Arg^30^ and Arg^35^ are independent.

Given the sum of our experiments reported here we conclude that PRMT5-catalyzed p65 methylation at Arg^174^, Arg^30^ and Arg^35^, is critical for p65 recruitment to the *CXCL11* promoter and the subsequent synergistic induction of *CXCL11* in response to TNF plus IFN-γ costimulation. This requirement does not exist for the synergistic induction of *CXCL10*. Our findings are consistent with a mechanism whereby concurrent stimulation of EC by both TNF and IFN-γ activate PRMT5. PRMT5 is then recruited to p65, where it catalyzes dimethylation of Arg^174^. Arginine methylation of p65 at Arg^174^ is necessary for p65 recruitment to the *CXCL11* promoter. Given the location of Arg^174^ on the p65 molecule is probable that modification of this residue facilitates a protein-protein interaction that enhances *CXCL11* transcription.

We do not currently know the mechanism linking activation of cytokine receptors with p65 methylation by PRMT5. One possible mechanism could involve activation of the JAK kinases by ligation of the IFN-γ receptor by IFN-γ. PRMT5 was first discovered in human cells as a JAK2 binding protein [50], and it may also interact with JAK1 and JAK3. JAK proteins have been shown to phosphorylate PRMT5 [51]. Phosphorylation of PRMT5 by a JAK may upregulate PRMT5 methyltransferase activity, or enhance it’s affinity for p65, leading to catalysis of SDMA formation at Arg^174^. TNF signaling would then mobilize the p65 methylated at Arg^174^ to the nucleus, where it associates with transcription cofactors and the *CXCL11* promoter. It is not clear whether IFN-γ alone is sufficient to trigger methylation at p65 at Arg^174^, or whether Arg^174^ methylation is a product of synergism between the TNF and IFN-γ pathways. We also can not rule out contribution of other PRMT enzymes in the methylation of Arg^174^, including PRMT1 and PRMT4, both of which are found in EC. PRMT1 and PRMT4 catalyze formation of ADMA, which may differ in transcriptional outcome to the SDMA modification catalyzed by PRMT5 that we report here.

Our findings suggest that the PRMT5-mediated methylation imparts an indexing signal to p65 that enables stimulus-specific chemokine expression. Differential combinations of such modifications enable p65 to interact with DNA or other components of specific transcriptional complexes in a context-specific manner, resulting in unique patterns of gene expression [22, 52]. Such a PTM code coupling extracellular stimuli to transcriptional outcomes is essential to the regulation of complex physiological and pathological processes such as inflammation. CXCL10 and CXCL11 are both found at high levels in human atheroma, as is TNF, and IFN-γ. CXCL10 and CXCL11 have redundant functions in that both ligate CXCR3 and promote chemotaxis of leukocytic populations and smooth muscle cells, and are angiostatic for EC [46, 53, 54]. Th1-type T-cells recruited by CXCL10 and CXCL11 exacerbate atherosclerosis by producing copious amounts of IFN-γ and proteinases that reduce collagen maturation [55]. However, CXCL10 and CXCL11 differ in their expression in diseases states such as atherosclerosis. Atheroma-associated EC, smooth muscle cells, and macrophages robustly produce CXCL10. CXCL11 is secreted by EC, macrophages, and lesional EC microvessels [56]. These cell types are found in discrete regions of the plaque, resulting in the accumulation of T-cells in the shoulder regions and fibrous cap [56, 57]. Varying levels of expression of both CXCL10 and CXCL11 by these cell types may be influenced by distinct regulatory and biochemical properties of these ligands. *CXCL10* expression is elicited by the widest variety of agonists, including IFN-α/β/γ, and is most sensitive to TNF. In contrast, *CXCL11* expression is weakly transcribed by TNF, but is induced by IFN-β/γ [33]. CXCL11 has greater potency than CXCL10 [34, 58, 59] as it is able to trigger receptor internalization, calcium mobilization, and chemotaxis at lower doses. These differences in CXCR3 ligand expression patterns according to cell type, agonist potency, and receptor affinity appear likely to exert fine control of T-cell recruitment within the lesion. Given our findings, the newly discovered specific PRMT5 inhibitors [60–62] should be investigated in animal models of chronic inflammatory disease as potential therapeutic agents to attenuate pathological progression.

## Supporting Information

S1 Fig NF-κB p65 is dimethylated at Arg^174^. MS/MS spectra of the 633 Da triply charged ion identified in the tryptic digestion of NF-κB p65. The mass of this peptide is consistent with the addition of two methyl groups to the ^172^PLRLPPVLSHPIFDNR^185^ peptide. This spectra contains several unmodified C-terminal y ions, all of which are consistent with dimethylation at Arg^174^.
(TIF)

S1 Table Details of antibodies used. (DOCX)

S2 Table Sequences of qRT-PCR primers for cDNA amplification. (DOCX)

S3 Table Sequences of qRT-PCR primers for ChIP promoter amplification. (DOCX)

## Abbreviations

PRMT5: protein arginine methyltransferase 5
p65: NF-κB p65 (RelA)
EC: endothelial cell
SDMA: symmetrical dimethylarginine
PCR: quantitative real-time
TNF: tumor necrosis factor-α
IFN: interferon-gamma (IFN-γ)
CXCL10: C-X-C motif chemokine 10 (IP-10)
CXCL11: C-X-C motif chemokine 11 (I-TAC)
CCL2: chemokine C-C motif ligand 2 (MCP-1)
CX3CL1: C-X3-C motif chemokine ligand 1 (fractalkine)
