# Supplementary figures and images for: PRMT5-Mediated Methylation of NF-κB p65 at Arg174 Is Required for Endothelial CXCL11 Gene Induction in Response to TNF-α and IFN-γ Costimulation
Source: PLoS One. 2016 Feb 22;11(2):e0148905. doi: 10.1371/journal.pone.0148905 (PMC4768879; doi:10.1371/journal.pone.0148905)

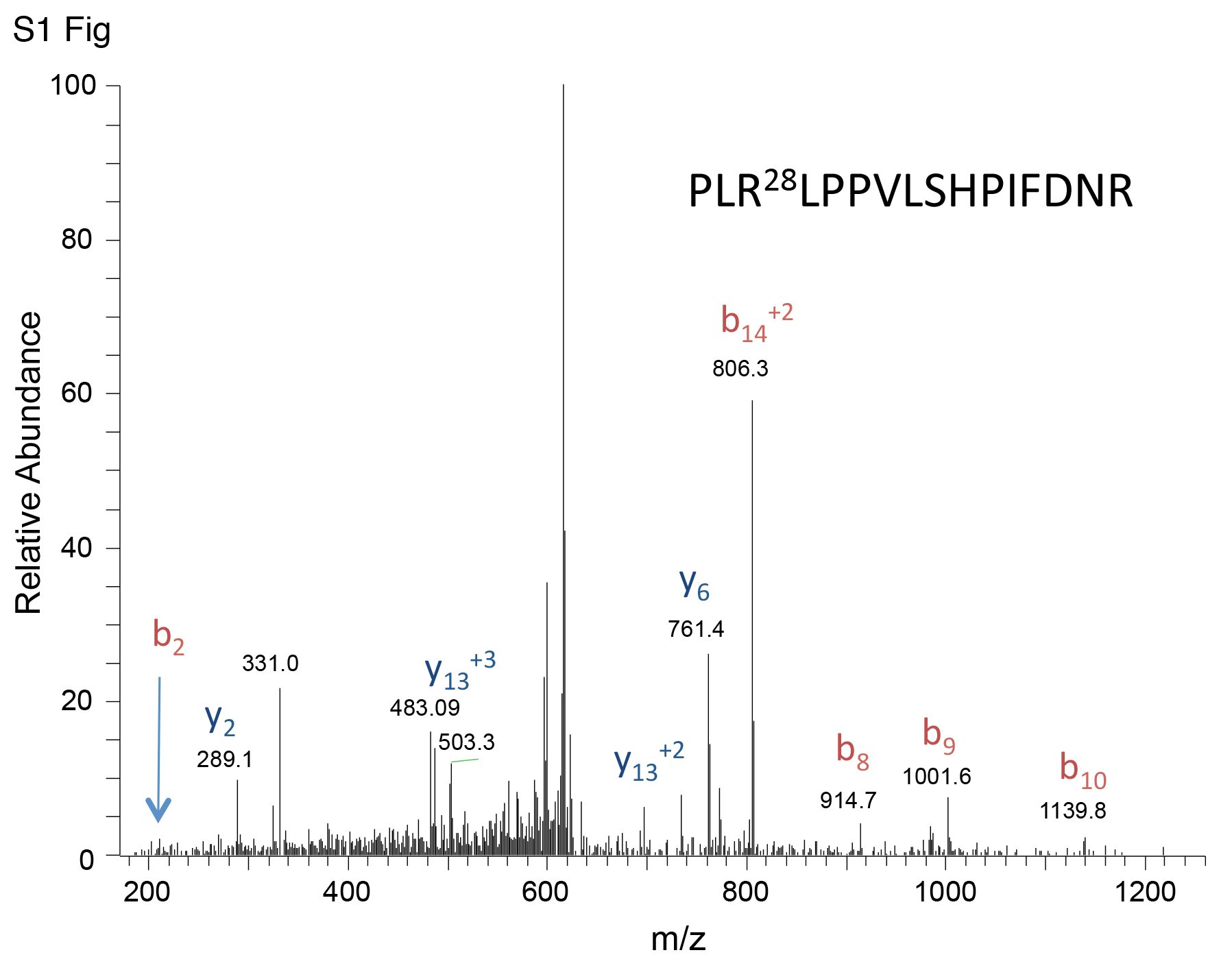

Supplement: S1 Fig — MS/MS spectra of the 633 Da triply charged ion identified in the tryptic digestion of NF-κB p65. The mass of this peptide is consistent with the addition of two methyl groups to the 172PLRLPPVLSHPIFDNR185 peptide. This spectra contains several unmodified C-terminal y ions, all of which are consistent with dimethylation at Arg174. (TIF) [file pone.0148905.s001.tif]
