# Supplementary material for: PRMT5-Mediated Methylation of NF-κB p65 at Arg174 Is Required for Endothelial CXCL11 Gene Induction in Response to TNF-α and IFN-γ Costimulation
Source: PLoS One. 2016 Feb 22;11(2):e0148905. doi: 10.1371/journal.pone.0148905 (PMC4768879; doi:10.1371/journal.pone.0148905)
Supplement: S1 Table — (DOCX) [file pone.0148905.s002.docx]

| **S1 Table.** List of antibodies for immunoblotting and immunoprecipitation. | |  |  |  |  |  |
| --- | --- | --- | --- | --- | --- | --- |
| **Antigen** | **Antibody Registry Identifier** | **Species** | **Clonality** | **App.** | **Conc.** | **Lot** |
| CXCL11 | R&D Systems Cat# MAB672, RRID:AB_2088019 | Mouse | Monoclonal | IB | 1:1000 | DYT0114021 |
| FLAG tag | Sigma-Aldrich Cat# P2983, RRID:AB_439685 | Mouse | Monoclonal | IP | 1:100 | SLK1346V |
| GAPDH | Cell Signaling Technology Cat# 2118L, RRID:AB_1031003 | Rabbit | Monoclonal | IB | 1:1000 | 8 |
| NF-kB p65 | EMD Millipore Cat# 06-418, RRID:AB_310117 | Rabbit | Polyclonal | IB | 1:1000 | L1911 |
| PRMT5 | Millipore Cat# 07-405, RRID:AB_310589 | Rabbit | Polyclonal | IB | 1:1000 | 2080918 |
| Symmetric | Millipore Cat# 07-412, RRID:AB_310594 | Rabbit | Polyclonal | IB | 1:1000 | 2510380 |
| Dimethylarginine |  |  |  |  |  |  |

**Legend:** App., Application; Conc., Concentration.
