# Supplementary material for: PRMT5-Mediated Methylation of NF-κB p65 at Arg174 Is Required for Endothelial CXCL11 Gene Induction in Response to TNF-α and IFN-γ Costimulation
Source: PLoS One. 2016 Feb 22;11(2):e0148905. doi: 10.1371/journal.pone.0148905 (PMC4768879; doi:10.1371/journal.pone.0148905)
Supplement: S2 Table — (DOCX) [file pone.0148905.s003.docx]

| **S2 Table.** qRT-PCR primers for cDNA amplification. | |
| --- | --- |
| **Template** | **Primer Sequence** |
| *CCL2/MCP-1* | F 5´-TCTCTTCCTCCACCACTAATGCA-3´ |
|  | R 5´-GGCTGAGACAGCACGTGGAT-3´ |
| *CX3CL1/Fractalkine* | F 5´-CATCATGCGGCAAACGCGCA-3´ |
|  | R 5´-AGCAGCCTGGCGGTCCAGAT-3´ |
| *CXCL8/IL-8* | F 5´-GGCCGTGGCTCTCTTGGCAG-3´ |
|  | R 5´-TGTGTTGGCGCAGTGTGGTCC-3´ |
| *CXCL10/IP-10* | F 5´-GCAGAGGAACCTCCAGTCTCAGCA-3´ |
|  | R 5´-GCTGATGCAGGTACAGCGTACAGT-3´ |
| *CXCL11/I-TAC* | F 5´-TGAGTGTGAAGGGCATGGCT-3´ |
|  | R 5´-GCTTTTACCCCAGGGCCTAT-3´ |
| *GAPDH* | F 5´-TCAACAGCGACACCCACTCC-3´ |
|  | R 5´-TGAGGT CCACCACCCTGTTG-3´ |
