# Supplementary material for: PRMT5-Mediated Methylation of NF-κB p65 at Arg174 Is Required for Endothelial CXCL11 Gene Induction in Response to TNF-α and IFN-γ Costimulation
Source: PLoS One. 2016 Feb 22;11(2):e0148905. doi: 10.1371/journal.pone.0148905 (PMC4768879; doi:10.1371/journal.pone.0148905)
Supplement: S3 Table — (DOCX) [file pone.0148905.s004.docx]

| **S3 Table.** qRT-PCR primers for ChIP promoter amplification. | |
| --- | --- |
| **Template** | **Sequence** |
| *CCL2* Promoter | F 5´-CCCATTTGCTCATTTGGTCTCAGC-3´ |
|  | R 5´-GCTGCTGTCTCTGCCTCTTATTGA-3´ |
| *CX3CL1* Promoter | F 5´-GGCATGTTCCCAGCTTGTGGCAGG-3´ |
|  | R 5´-GTTGCCAAGGAACCAAGCCGGC-3´ |
| *CXCL10* Promoter | F 5´-AGGAGCAGAGGGAAATTCCGTAAC-3´ |
|  | R 5´-AACGTGGGGCTAGTGTGCCA-3´ |
| *CXCL11* Promoter | F 5´-TGGAAGCAGGAAAGGTGCAT-3´ |
|  | R 5´-TGGAAGGAGTAGAAATGCTGAACA-3´ |
